# HPV-Associated Oropharyngeal Squamous Cell Carcinoma and Persistent Oral High-Risk HPV Detection: A Narrative Review of Longitudinal Assessment, Diagnostic Evaluation, and Surveillance

**DOI:** 10.3390/ijms27188411

**Published:** 2026-09-21

**Authors:** Aornrutai Promsong, Wipawee Nittayananta

**Affiliations:** 1 Faculty of Medicine, Princess of Naradhiwas University, Narathiwat 96000, Thailand; 2 Faculty of Dentistry, Bangkokthonburi University, Bangkok 10170, Thailand

**Keywords:** human papillomavirus, oral HPV, persistent oral HPV detection, oropharyngeal squamous cell carcinoma

## Abstract

Currently, no validated population-based screening strategy uses persistent oral high-risk human papillomavirus (HPV) detection to identify individuals at clinically meaningful risk of HPV-associated oropharyngeal squamous cell carcinoma (OPSCC). This narrative review evaluates sampling strategies, virologic assays, liquid-biopsy biomarkers, adjunct biomarkers, and integrative approaches across three clinical contexts: longitudinal oral HPV assessment; diagnostic evaluation and HPV attribution in suspected or newly diagnosed OPSCC; and treatment monitoring and post-treatment surveillance in established HPV-associated OPSCC. The literature published from January 2020 through June 2026 and identified in PubMed was synthesized by clinical context, specimen source, biological target, study population, and intended use. Repeated detection of the same high-risk HPV genotype in serial oral specimens provides a pragmatic measure of persistent oral HPV detection but does not establish uninterrupted infection, sustained viral oncogene transcription, malignant transformation, or future OPSCC risk. Oral HPV DNA, oral E6/E7 mRNA, tumor-tissue HPV testing, and plasma circulating tumor HPV DNA represent distinct biological and clinical signals. Plasma circulating tumor HPV DNA has the most mature evidence for treatment monitoring and post-treatment surveillance in established HPV-associated OPSCC, not population screening. Clinical translation requires standardized longitudinal protocols, validation in intended-use populations, actionable management pathways, and incremental predictive value for proposed risk-stratification models.

## 1. Introduction

Among head and neck squamous cell carcinomas (HNSCCs), oropharyngeal squamous cell carcinoma (OPSCC) is anatomically distinct and includes human papillomavirus (HPV)-associated and HPV-unrelated forms that differ in etiology and biology. High-risk HPV is an established causal driver of HPV-associated OPSCC, although the proportion of OPSCC attributable to HPV varies geographically [1,2]. Population-based studies have generally shown increasing incidence at head and neck cancer subsites classified as HPV-related, alongside declining or stable incidence at most HPV-unrelated subsites; however, these classifications are anatomical proxies and do not directly establish tumor-level HPV status [3]. Consistent with this epidemiologic shift, an international registry analysis of 42 countries found significant increases in oropharyngeal cancer incidence in 19 countries among men and 23 among women, with incidence rates approximately three- to fourfold higher in men in most countries. These patterns varied substantially by country, sex, and age [4]. In men, the observed trends were largely consistent with an increasing HPV-associated burden, whereas patterns among women were more heterogeneous, suggesting varying contributions of HPV and traditional risk factors across countries [4]. Among high-risk HPV genotypes detected in oropharyngeal cancer, HPV16 predominates, whereas HPV33, HPV18, HPV35, and HPV58 are much less common [5].

HPV-associated OPSCC arises predominantly in the lymphoepithelial tissue of the palatine and lingual tonsils, where the deeply invaginated crypt architecture can limit direct visualization of early or occult lesions [1,6]. A cryptal origin is not universal. Recent evidence from a systematic review and spatial transcriptomic analysis suggests that a subset of HPV-associated tonsillar squamous cell carcinomas may arise from the tonsillar surface and progress from dysplasia to invasive disease [7]. The implications of these findings for screening, however, remain uncertain. Unlike cervical cancer, HPV-associated OPSCC currently lacks a clinically recognized and validated precursor lesion suitable for screening, and no validated population-based screening strategy has been established [6,7].

The central challenge is therefore not simply detecting HPV but determining the biological and clinical significance of a positive result. HPV DNA detected in an oral rinse, gargle, or saliva specimen does not by itself identify the anatomical source of the viral material, establish how long HPV has been present, demonstrate viral oncogene transcription or malignant transformation, or provide a validated estimate of future OPSCC risk [6,8,9,10]. Longitudinal estimates of oral HPV persistence and clearance are also sensitive to sampling interval and the operational definitions used [8,9]. Repeated detection of the same high-risk HPV genotype therefore provides a more informative longitudinal endpoint than a single positive result, but it remains an observed detection pattern rather than evidence of uninterrupted infection [8,9]. Viral E6/E7 transcription and tumor-derived HPV signals provide distinct biological and clinical information and should not be considered interchangeable with mucosal HPV detection. Their detection should also not be taken to establish a validated linear progression from oral HPV detection to OPSCC [6,10].

Recent reviews have separately addressed laboratory HPV testing in oral and blood specimens, surrogate biomarkers for the early detection of HPV-associated OPSCC, liquid biopsy approaches using saliva and blood, and diagnostic and emerging molecular methods for HPV detection in OPSCC [6,11,12,13]. Against this background, this narrative review integrates these evidence streams across three distinct clinical contexts: longitudinal oral HPV assessment in individuals without known OPSCC; diagnostic evaluation and HPV attribution in suspected or newly diagnosed OPSCC; and treatment response assessment and post-treatment surveillance in patients with established HPV-associated OPSCC. Within these contexts, evidence is interpreted according to specimen source, biological target, and intended clinical use, while analytical platforms are considered cross-cutting methods for detecting or quantifying these targets. Particular emphasis is placed on distinguishing mucosal HPV detection, repeated type-specific detection, viral oncogene transcription, and tumor-derived HPV signals, which represent biologically and clinically distinct forms of evidence and should not be interpreted interchangeably. The review further distinguishes analytical performance from clinical validity and clinical utility and considers the maturity of evidence supporting clinical interpretation or application. It examines sampling and pre-analytical factors, virologic assays, oral-fluid biomarkers, plasma circulating tumor HPV DNA, candidate adjunct biomarkers, and emerging discovery and integrative technologies. Particular attention is given to the directness of the evidence, the populations and clinical contexts in which each approach has been evaluated, and whether the findings support longitudinal oral HPV assessment, prediagnostic OPSCC risk prediction, diagnostic HPV attribution, treatment monitoring, post-treatment surveillance, or future clinical translation.

## 2. Literature Search and Review Approach

This narrative review focused on the literature published from January 2020 through June 2026 concerning longitudinal oral and oropharyngeal HPV assessment, HPV-associated OPSCC, and established and emerging sampling and biomarker approaches. The review focused on this period to emphasize contemporary evidence relevant to current sampling methods and molecular, liquid-biopsy, and computational approaches. PubMed was the sole bibliographic database used to identify the relevant literature. Searches were conducted using the PubMed Advanced Search Builder by combining Medical Subject Headings (MeSH) and Title/Abstract terms with the Boolean operators AND and OR. Search concepts included oral and oropharyngeal HPV persistence and clearance, oral and oropharyngeal sampling, HPV DNA and E6/E7 mRNA, liquid biopsy, circulating tumor HPV DNA, candidate biomarkers, and emerging molecular and computational approaches. Because this was a narrative rather than a systematic review, studies were selected for their relevance to the biological or clinical questions addressed rather than through a formal systematic-review screening process. Primary studies were prioritized when study-specific methodological, analytical, or numerical findings were required, whereas systematic reviews, meta-analyses, and relevant clinical or pathological guidelines were used primarily for broader evidence synthesis and clinical context. Studies involving broader head and neck squamous cell carcinoma populations, non-OPSCC disease, HPV-negative tumors, or preclinical models were considered only when they directly informed the methodological, biological, or discovery-stage concepts discussed in this review. Such evidence was interpreted within its original study population and intended use and was not assumed to be directly transferable to longitudinal oral HPV assessment or prediagnostic OPSCC risk prediction in individuals without known OPSCC. Interpretation considered population characteristics, specimen type, sampling interval, genotype-specific assessment, study design, clinical setting, and whether the evidence was derived from cancer-free populations, patients with suspected or newly diagnosed disease, or patients with established cancer. Because the available studies differed substantially in population, specimen type, sampling interval, assay platform, definitions of persistence and clearance, and clinical endpoints, findings were synthesized narratively rather than quantitatively. The synthesis was organized primarily by clinical context and intended use and secondarily by specimen source and biological target. Analytical platforms were interpreted as cross-cutting methods for detecting or quantifying biological targets rather than as independent biomarkers. The synthesis specifically distinguished mucosal HPV detection, repeated type-specific detection, viral oncogene transcription, and tumor-derived HPV signals.

For interpretation across studies, analytical performance, clinical validity, and clinical utility were considered separately. Analytical performance referred to assay characteristics for detecting or quantifying the intended molecular target within the evaluated specimen and assay context, including analytical sensitivity, analytical specificity, reproducibility, and limits of detection or quantification when reported. Clinical validity referred to the extent to which a detected signal was associated with or predictive of a clinically relevant disease state or outcome. Clinical utility referred to evidence that test-guided management provides clinically meaningful benefit relative to an appropriate alternative, taking potential harms and burdens into account. Increased analytical sensitivity or detection yield was therefore not interpreted as evidence of clinical validity or utility in the absence of corresponding clinical evidence. The clinical evidence-maturity categories used in this review were qualitative and were intended to describe the level of evidence supporting clinical interpretation or application rather than to constitute a formal Grading of Recommendations Assessment, Development and Evaluation (GRADE) assessment. “Established” denoted guideline-supported clinical use within a defined indication; “clinically advanced” denoted substantial human evidence with remaining limitations in routine implementation or management pathways; “candidate” denoted human evidence supporting a biologically or clinically relevant interpretation for a specified intended use but without sufficient validation for routine clinical use; “exploratory” denoted early human association or feasibility evidence without sufficient clinical validation for the intended use; and “discovery-stage” denoted evidence directed primarily toward mechanistic characterization, biomarker discovery, or model development without sufficient clinical validation for the intended use. The use of high-dimensional data alone did not determine evidence maturity. When evidence could plausibly fit more than one category, classification reflected the most mature level supported by the available evidence for the specific specimen, study population, and intended use.

## 3. Longitudinal Oral HPV Assessment: Operational Definitions, Sampling, and Pre-Analytical Considerations

### 3.1. Operational Definition and Methodological Heterogeneity

Longitudinal studies have operationalized oral HPV persistence and clearance using heterogeneous definitions and analytical approaches (Table 1). Cohorts differ in specimen type and sampling schedule [8,14,15,16], while type-specific assessment has been implemented using different criteria for the number and sequence of positive or negative visits and, in some studies, the handling of intervening negative results [8,9,14,16,17]. Interpretation also depends on whether infections were prevalent at baseline or newly detected during follow-up [8,9,17] and on the analytical approach used to estimate HPV dynamics, including multistate transition modeling in some cohorts [15,16]. In the Persistent Oral Papillomavirus Study (POPS), participants underwent oral rinse and gargle sampling approximately every six months for four years, and clearance was primarily defined as two consecutive type-specific negative tests. Subsequent long-term follow-up included participants enrolled in the Men and Women Understanding Throat HPV (MOUTH) study [8]. A later analysis evaluated clearance using one, two, or three consecutive negative results, as well as negativity at the final two visits, and showed that estimated time to clearance varied substantially according to the definition used [9]. In contrast, the Michigan HPV and Oropharyngeal Cancer Study used follow-up intervals of approximately 3–4 months and a multistate transition model, which estimated a mean time to clearance of a previously detected oral HPV genotype of 46 days [15]. Direct comparison of these estimates requires caution because the studies differed in population characteristics, sampling schedules, analytical methods, and statistical approaches.

Genotype resolution is important for evaluating type-specific persistence because repeated HPV positivity without genotyping cannot establish repeated detection of the same HPV genotype. Even repeated type-specific detection, however, does not demonstrate uninterrupted biological persistence. Intervening negative results may reflect sampling limitations or fluctuations near the assay detection limit, while true clearance followed by reacquisition of the same genotype is also possible; available longitudinal data cannot reliably distinguish among these explanations [9]. The handling of intermittent negativity also differs across studies. In the HPV Infection in Men (HIM) Study, persistence of an individual HPV genotype was generally assessed by repeated detection at consecutive visits approximately six months apart. In a small number of cases, an intervening genotype-specific negative result was retained within the persistence classification when the same genotype was detected at the preceding visit and again at the next two visits [14].

For this review, “persistent oral HPV detection” refers pragmatically to repeated detection of the same high-risk HPV genotype in serial oral specimens. This term describes an observed longitudinal detection pattern rather than a confirmed biological state. It should not be taken as evidence of uninterrupted infection, active viral replication, sustained E6/E7 oncogene transcription, or malignant transformation, and it has not been validated for individual-level prediction of future OPSCC. Cross-study comparisons should therefore consider sampling interval, number of observations, genotype concordance, clearance criteria, handling of intermittent negative results, statistical approach, and whether HPV was prevalent or newly detected during follow-up.

### 3.2. Oral Rinse and Gargle Sampling

Oral rinse and gargle are practical, noninvasive methods for sampling the oral cavity and oropharynx and are widely used in epidemiological and research settings [11]. Serial rinse-and-gargle specimens have also been used in longitudinal oral HPV studies [8]. For clarity, in this review, oral rinse refers to swishing a collection fluid throughout the oral cavity, whereas gargle refers to agitating the fluid in the posterior oral cavity and oropharynx. These maneuvers are commonly performed sequentially as a single collection procedure [11]. Because the resulting specimen is non-targeted, it does not provide site-specific localization of detected HPV DNA [18].

Oral HPV sampling protocols remain heterogeneous across studies [11]. In POPS, oral rinse and gargle specimens were collected using 10 mL of saline or Scope mouthwash for 30 s [8]. Castaneda-Avila et al. used 10 mL of Scope (original mint flavor) for a 30-s self-collected rinse and gargle under study supervision [19], whereas the Oromouth study used a 60-s oral rinse and gargle with sterile saline [20]. These examples illustrate variation in collection medium, duration, and procedural details. Such pre-analytical variables should be reported when comparing HPV detection across studies [11]. Precollection oral behaviors are another protocol consideration. Castaneda-Avila et al. recorded the time since last tooth brushing and mouthwash use on the day of sampling, but these variables were not evaluated to determine an optimal precollection interval for oral HPV testing [19]. No standardized HPV-specific precollection abstinence interval for eating, drinking, smoking, tooth brushing, or mouthwash use was identified in the studies reviewed here. Longitudinal protocols should therefore prespecify and document relevant precollection conditions to improve reproducibility.

Postcollection handling is another important component of the pre-analytical workflow. In POPS, oral rinse specimens were stored at 4 °C until processing [8]. Immediate addition of a preservative may improve oral-specimen stability. In the absence of a preservative, prompt processing or immediate freezing at −20 °C has been recommended to minimize degradation. However, the effect of storage time, including frozen storage, on sample quality and target deterioration remains uncertain [11]. Longitudinal studies should therefore prespecify and report the interval from collection to processing, storage temperature and duration, preservative use, and subsequent processing and nucleic acid extraction procedures. Harmonization and transparent reporting of these pre-analytical steps are important for reproducible serial HPV assessment.

Taken together, oral rinse and gargle are practical broad-field methods for longitudinal oral HPV research, but their interpretation depends on consistent collection and pre-analytical handling. Serial HPV findings should be interpreted according to the operational definition of persistent oral HPV detection in Section 3.1. Their use in longitudinal research should be distinguished from site-directed sampling and from population screening; no validated population-based screening strategy for HPV-associated OPSCC is currently available [6,11].

### 3.3. Site-Directed Oral and Oropharyngeal Sampling

The predilection of HPV-associated OPSCC for the palatine and lingual tonsils provides an anatomical rationale for site-directed sampling, particularly because these tumors commonly involve the specialized reticulated epithelium of the tonsillar crypts [1,6]. This anatomical model is not universal. A recent systematic review and meta-analysis found that a subset of HPV-associated tonsillar squamous cell carcinomas may arise from the tonsillar surface, while complementary spatial transcriptomic and trajectory analyses supported a progression from dysplasia to invasive disease [7]. These findings broaden the anatomical model of HPV-associated tonsillar carcinogenesis but do not establish the performance of any sampling method. Anatomical plausibility should therefore be distinguished from evidence that current in vivo approaches reliably access the relevant epithelial compartments.

Comparative evidence on oral HPV sampling methods remains limited [18]. In the Oromouth cohort, oral rinse had the highest individual high-risk HPV detection rate, although sampling of the pharyngeal wall, base of tongue, and tonsillar tissue identified additional high-risk HPV-positive findings not detected by oral rinse alone [20]. The tonsillar specimens, however, were surgically obtained at tonsillectomy, and the sampling comparison was based on specimens collected at study entry. These findings therefore inform cross-sectional detection across sampling fields but do not address persistent type-specific HPV detection or subsequent OPSCC risk. In 435 healthy Japanese participants, adding tonsillar washing after oral gargling produced only a small, statistically non-significant increase in HPV detection [21]. Although the procedure was designed to enhance sampling of the tonsillar region, the study did not establish reproducible sampling of the deep tonsillar crypt epithelium.

Taken together, these studies show that additional oropharyngeal sampling can alter cross-sectional HPV detection yield, but greater aggregate detection should not be assumed to confer greater clinical validity. It remains unclear which sampling method best represents clinically relevant prevalent or persistent oral HPV detection [18], and current evidence does not establish that site-directed brushing or washing improves longitudinal identification of persistent type-specific oral HPV or predicts subsequent HPV-associated OPSCC. Site-directed sampling should therefore currently be regarded as investigational for longitudinal oral HPV risk assessment. Future prospective studies should compare standardized, clinically feasible site-directed approaches with oral rinse or gargle using serial genotype-specific assessment and determine whether incremental detection is reproducible and associated with clinically relevant longitudinal outcomes.

## 4. Virologic Detection and Analytical Platforms

### 4.1. HPV DNA Detection and Genotype-Specific Testing

HPV DNA assays detect viral genomic sequences and, depending on assay design, can provide broad-spectrum HPV detection, genotype-specific identification, and quantitative estimates of viral DNA in oral-fluid, site-directed mucosal, and tissue specimens. Polymerase chain reaction (PCR)-based genotyping has been used in longitudinal oral HPV cohorts to track individual HPV types across serial specimens [8,17]. When the longitudinal endpoint is repeated detection of the same HPV type, genotype resolution is essential because serial HPV positivity without type information cannot establish genotype concordance across visits. Analytical performance varies with specimen matrix, pre-analytical handling, genomic target, genotype coverage, assay platform, and detection threshold [11,22]. Quantitative PCR, droplet digital PCR, and next-generation sequencing are discussed separately in Section 4.3 and Section 4.4.

In oral-fluid specimens, a positive HPV DNA result establishes only that viral DNA is detectable in the collected specimen. It does not localize the anatomical source, determine the duration of detection, demonstrate viral oncogene transcription or malignant transformation, or by itself establish persistent oral HPV detection as defined in Section 3.1 [6,11]. In one longitudinal cohort, lower HPV16 viral load was associated with a greater likelihood of clearance, but the study did not define or validate a clinically actionable viral-load threshold [8]. Viral-load measurements and increased analytical sensitivity should therefore not be equated with clinical validity or clinical utility. Oral HPV DNA testing has not been validated as a stand-alone population screening test or as an individual-level predictor of incident HPV-associated OPSCC [6,9,11].

HPV testing in tumor tissue addresses a different clinical question from oral-fluid testing: determining HPV status in an established carcinoma. Detection of HPV DNA confirms the presence of viral DNA but does not directly establish viral oncogene transcription. The 2025 College of American Pathologists guideline recommends high-risk HPV testing for all patients with newly diagnosed OPSCC, regardless of histologic subtype [2]. For noncytology oropharyngeal specimens, including regional nodal metastases when an oropharyngeal primary is clinically supported, p16 immunohistochemistry is recommended as the surrogate test. p16 positivity is defined as nuclear and cytoplasmic staining of at least moderate intensity in 70% or more of tumor cells. HPV-specific testing is recommended in selected settings, including equivocal p16 staining, discordance between p16 staining and tumor morphology, regions with a low prevalence of HPV-associated OPSCC, large multisite tumors involving the oropharynx, specimens from oropharyngeal sites other than the tonsil or base of tongue, and cases in which HPV-specific testing is required by a clinical trial [2]. Metastatic squamous cell carcinoma of unknown primary in a cervical lymph node follows a separate testing algorithm and should not be subsumed under this p16-only framework. This tissue-based diagnostic approach should not be extrapolated to oral-fluid HPV DNA testing in individuals without known OPSCC. E6/E7 mRNA testing, which addresses viral oncogene transcription rather than the presence of viral DNA, is discussed separately in Section 4.2.

### 4.2. Viral Oncogene Transcription: E6/E7 mRNA

E6/E7 mRNA assays detect viral oncogene transcripts and therefore provide evidence of HPV transcription rather than simply the presence of viral DNA. In tumor tissue, detection of E6/E7 mRNA provides direct evidence of transcriptionally active HPV and has been used as a reference standard for transcriptional activity. RNA in situ hybridization (RNA-ISH) additionally localizes viral transcripts within tissue and is among the HPV-specific assays recommended when confirmatory testing is indicated. DNA PCR may also be used but should be interpreted with p16 immunohistochemistry, whereas reverse-transcription PCR for E6/E7 mRNA is less practical for routine tissue testing because of technical complexity and challenges in defining positivity thresholds [2]. Thus, E6/E7 mRNA provides more direct biological evidence of viral oncogene transcription than HPV DNA detection, but this distinction does not imply universal superiority in analytical or diagnostic performance [2,10].

Evidence from oral-fluid specimens is more limited. Among patients with high-risk HPV-associated OPSCC, Tanaka et al. reported sensitivities of 82% for oral HPV mRNA and 80% for oral HPV DNA, with specificities of 94% and 100%, respectively. Performance was substantially lower in the small HPV16-related squamous cell carcinoma of unknown primary subgroup, indicating that oral-assay performance depends on clinical presentation and anatomical context. Assay genotype coverage also affected detection: both HPV69-related OPSCCs were negative because HPV69 was outside the target range of the oral assays, while the Aptima assay did not provide genotype-specific results [10]. In saliva, Deutsch et al. demonstrated that HPV16 E6/E7 transcripts could be detected using biplex reverse-transcription quantitative PCR. However, the saliva cohort comprised only eight patients, the assay was restricted to HPV16, and formal analyses of the limit of detection and limit of quantification were not performed [23]. This study supports technical feasibility but does not establish diagnostic performance, longitudinal reproducibility, or screening utility.

Overall, oral-fluid E6/E7 mRNA provides biologically distinct information from HPV DNA by demonstrating viral oncogene transcripts in the collected material, but it does not establish their anatomical source [11]. Current evidence derives mainly from patients with established cancer and does not establish its role in longitudinal risk assessment in individuals without known OPSCC [10,23]. Prospective studies are needed to determine whether serial E6/E7 mRNA assessment adds clinically meaningful information beyond standardized genotype-specific HPV DNA testing.

### 4.3. Quantitative Real-Time PCR and Droplet Digital PCR

Quantitative real-time PCR (qPCR) and droplet digital PCR (ddPCR) are targeted platforms used to detect and quantify HPV DNA. qPCR measures fluorescence during amplification and typically estimates target abundance against a calibration curve, whereas ddPCR partitions the reaction into droplets and estimates absolute target concentration from endpoint detection using Poisson-based analysis without an external standard curve. Both platforms can support genotype-specific detection, but their analytical performance depends on specimen matrix, target design, genotype coverage, nucleic acid input, pre-analytical handling, and the threshold used to define positivity [22,24]. Performance should therefore be interpreted within the specimen type and clinical setting in which the assay was evaluated.

Comparative clinical evidence derives mainly from studies of pretreatment circulating tumor HPV DNA (ctHPV DNA) in blood samples from patients with established HPV-associated cancers. In a meta-analysis restricted to HPV-associated OPSCC, pooled sensitivity for ctHPV DNA detection was 0.89 for ddPCR and 0.66 for qPCR, with high specificity for both platforms [25]. These estimates represent clinical detection performance across heterogeneous studies rather than intrinsic analytical sensitivity. Specimen dependence was also evident in a direct comparison of patients with HPV-associated OPSCC: ddPCR and qPCR detected HPV16 DNA more frequently in plasma than in oral rinse, whereas next-generation sequencing showed substantially greater sensitivity in oral rinse [22]. Thus, ddPCR should not be considered universally superior to qPCR or other molecular platforms. Clinical applications of plasma ctHPV DNA are discussed separately in Section 5.

Genotype coverage and matrix-specific validation are also important. A multiplex ddPCR assay was developed and analytically validated in platelet-poor plasma for detection and quantitation of ctHPV DNA from HPV16, HPV18, HPV31, HPV33, and HPV35, including assessment of precision, limits of detection and quantitation, analytical measuring interval, and analytical specificity [24]. These performance characteristics should not be generalized to oral specimens. In longitudinal oral HPV research, D’Souza et al. used quantitative PCR to assess HPV16 viral load and found that lower viral load was associated with a greater likelihood of clearance, but the study did not establish a clinically actionable threshold [8]. Evidence for ddPCR in the literature reviewed here relates predominantly to cancer-associated plasma ctHPV DNA rather than longitudinal oral HPV assessment in individuals without known OPSCC. qPCR and ddPCR should therefore be interpreted as context-dependent analytical platforms rather than independent biomarkers of persistent oral HPV detection or future OPSCC risk. Their clinical interpretation depends on the specimen matrix, biological target, assay design, and intended use.

### 4.4. Next-Generation Sequencing

Next-generation sequencing (NGS) comprises analytically distinct strategies, including targeted viral sequencing, tumor genomic panels, whole-genome sequencing, and RNA sequencing. Targeted viral sequencing can provide genotype-resolved HPV detection and viral sequence information, whereas tissue-based genomic and transcriptomic sequencing can characterize virus–host integration, host genomic alterations, and transcriptional profiles. These applications address different biological and clinical questions [26]. In a whole-genome sequencing analysis of 51 HPV-positive HNSCC tumors, 44% of evaluable integration breakpoints were subclonal, and at least 49% of tumors lacked clonal HPV integration. These findings demonstrate substantial heterogeneity in HPV integration and indicate that clonal integration is not required in all HPV-associated tumors [27]. However, integration status alone does not establish viral oncogene transcription or persistent oral HPV detection.

Clinical detection evidence summarized here derives mainly from patients with established HPV-associated cancer. In HPV-associated OPSCC, NGS was substantially more sensitive than ddPCR or qPCR for HPV16 DNA detection in oral rinse, whereas NGS and ddPCR showed similar sensitivity in plasma [22]. A meta-analysis of pretreatment blood-based ctHPV DNA studies reported pooled sensitivities of 0.91 for NGS, 0.89 for ddPCR, and 0.66 for qPCR in HPV-associated OPSCC; NGS did not significantly outperform ddPCR [25]. These findings show that NGS performance varies with specimen matrix and assay design and does not establish universal superiority over other molecular platforms. They should not be extrapolated to screening or longitudinal assessment in individuals without known OPSCC. Clinical applications of plasma ctHPV DNA are discussed separately in Section 5.

For longitudinal oral HPV research, the potential value of NGS lies in genotype and sequence resolution, including detection of multiple HPV types and intratype variation. Within the literature reviewed here, no NGS-based approach has been clinically validated for longitudinal prediction of incident HPV-associated OPSCC in individuals without known disease. NGS should therefore be regarded as a context-dependent analytical and research platform, with interpretation determined by specimen type, biological target, assay design, and intended use.

## 5. Liquid Biopsy Biomarkers Across Clinical Contexts

Liquid biopsy enables the molecular analysis of cellular and acellular analytes in biofluids, but interpretation depends on specimen source, biological target, and intended clinical use. In HPV-associated OPSCC, oral-fluid biomarkers may reflect mucosal HPV detection, epithelial shedding, or locally shed tumor-associated material, whereas plasma ctHPV DNA in patients with established disease is primarily interpreted as a tumor-derived circulating signal. These biomarkers therefore provide different biological and clinical information and should not be interpreted interchangeably [12,28].

### 5.1. Oral-Fluid Biomarkers in Longitudinal Oral HPV Assessment

Saliva, oral rinse, and gargle permit repeated noninvasive sampling for oral HPV assessment [8,11]. These specimens may contain viral nucleic acids within cellular material, while saliva has also been investigated for cell-free DNA (cfDNA) and analytes associated with extracellular vesicles [29,30]. The analytical performance of assays applied to these specimens depends on specimen collection, cellularity, preservation, processing, nucleic acid recovery, and assay design [11,29]. HPV DNA detection in oral specimens and variation in molecular platform performance across specimen matrices are discussed in Section 4. Brooks et al. optimized methods for recovering short salivary cfDNA fragments and detected HPV16 DNA in saliva from all three pretreatment patients with HPV-associated OPSCC included in the study. These findings support methodological feasibility but do not establish clinical validity for longitudinal oral HPV assessment or screening [29].

Salivary E6/E7 mRNA evidence from the small feasibility study reviewed here remains exploratory [23]. However, when considered alongside broader oral-fluid E6/E7 mRNA evidence from patients with known or suspected cancer [10,23], the overall evidence for oral-fluid E6/E7 mRNA is classified as candidate. Exosomal miRNA profiles in saliva have also differed between patients with HNSCC and healthy controls. However, the supporting study evaluated patients with established HNSCC and tumor HPV status rather than longitudinal persistent oral HPV detection [30]. These oral-fluid analytes therefore remain investigational for longitudinal oral HPV assessment and have not been validated to classify persistent type-specific oral HPV detection or predict incident HPV-associated OPSCC in individuals without known cancer [6,11].

### 5.2. Plasma ctHPV DNA and Other Circulating Biomarkers

Plasma ctHPV DNA has been investigated primarily as a tumor-derived biomarker in patients with established HPV-associated OPSCC, particularly for treatment response assessment, molecular residual disease assessment, and post-treatment surveillance [31,32]. Commercially available and laboratory-developed plasma ctHPV DNA assays differ in analytical architecture, genotype coverage, validation framework, and intended use (Table 2). Reported performance estimates are therefore not directly interchangeable because studies differ in disease setting, sampling time, reference standard, assay design, and clinical endpoint. In the corrected analysis of a prospective surveillance cohort, eight of 45 patients had at least one positive post-treatment ctHPV DNA result, of whom four developed recurrences confirmed by biopsy. All four patients with two consecutive positive tests developed recurrence, whereas none of the remaining 41 patients did [31]. The criterion of two consecutive positive tests was not prespecified and therefore requires independent prospective validation [31]. The median lead time from the first positive test to confirmation of recurrence by biopsy was 6.6 months [31]. Although these findings support the potential value of serial ctHPV DNA assessment for recurrence surveillance, they were based on only four recurrence events, and an isolated positive result was therefore not definitive evidence of recurrent disease.

A subsequent prospective surveillance study using a tumor tissue-modified viral HPV DNA (TTMV-HPV DNA) assay reported both recurrences with undetectable TTMV-HPV DNA at the time of recurrence diagnosis and prolonged detectable results without documented disease [34]. These observations show that ctHPV DNA surveillance may produce undetectable results despite documented recurrence as well as prolonged detectable results without confirmed disease and should therefore be interpreted within established clinical evaluation pathways rather than in isolation. A separate study in a broader HPV-associated HNSCC population similarly associated persistent post-treatment circulating tumor DNA (ctDNA) with residual or recurrent disease, although only four patients had positive post-treatment ctDNA and the cohort was not restricted to OPSCC [39]. ReACT 1.0 extended ctHPV DNA investigation from disease monitoring to biomarker-informed treatment adaptation. In this exploratory, nonrandomized phase II trial, 29 patients initially classified as being at clinically intermediate risk met prespecified TTMV-HPV DNA criteria, were reclassified as low risk, and received treatment deintensification. Progression-free survival at two years was 92% (95% CI, 83–100) [40]. These findings demonstrate the feasibility of incorporating ctHPV DNA kinetics into treatment-stratification research but do not establish comparative effectiveness or routine clinical utility.

Importantly, a biomarker signal preceding clinically confirmed recurrence and the feasibility of biomarker-informed treatment adaptation should not be equated with demonstrated improvement in patient outcomes. Establishing clinical utility requires evidence that biomarker-guided management provides clinically meaningful benefit compared with an appropriate standard-management strategy. Among the liquid-biopsy biomarkers considered here, plasma ctHPV DNA has the most mature clinical evidence in established HPV-associated OPSCC, particularly for disease monitoring and post-treatment surveillance [41]. However, changes to routine management based on biomarker results require prospective comparative evaluation, clinically actionable thresholds, and validated management pathways. Other circulating analytes, including nonviral cfDNA alterations, circulating tumor cells, extracellular vesicles, microRNAs, and methylation signatures, remain at earlier stages of clinical development, and their incremental value beyond ctHPV DNA has not been established [28,41].

### 5.3. Context-Specific Interpretation and Clinical Utility

Liquid-biopsy signals should be interpreted according to their biological source, study population, and intended clinical use. Oral HPV DNA indicates viral DNA detected in an oral specimen, whereas oral E6/E7 mRNA provides evidence of viral oncogene transcription in the sampled material [10,11]. In patients with established HPV-associated OPSCC, plasma ctHPV DNA represents a tumor-derived circulating signal that has been investigated primarily for disease assessment, treatment monitoring, and recurrence surveillance [41]. These signals are biologically and clinically distinct and should not be interpreted interchangeably. An isolated oral HPV DNA-positive result does not define persistent oral HPV detection, which requires repeated type-specific detection in serial oral specimens [9]. Likewise, plasma ctHPV DNA positivity should not be interpreted as evidence of persistent oral HPV detection.

For individuals without known OPSCC, repeated genotype-specific HPV detection in serial oral specimens provides the pragmatic longitudinal virological endpoint used in this review, as defined in Section 3.1 [9]. Current evidence reviewed here has not established clinical utility for population screening using biomarkers in oral fluid or blood, nor has it established a validated approach for individual-level prediction of incident HPV-associated OPSCC [6,12]. Future targeted risk-stratification approaches, if developed, would likewise require prospective validation in populations representative of their intended use and should not be assumed to have clinical utility solely because they identify molecular or virological abnormalities. In established HPV-associated OPSCC, plasma ctHPV DNA has prospective evidence for post-treatment recurrence surveillance and exploratory evidence for biomarker-informed treatment adaptation [31,34,40]. However, surveillance performance, earlier biomarker detection, and clinical utility represent distinct levels of evidence. A biomarker may become positive before conventional confirmation of recurrence without demonstrating that management based on that earlier signal improves patient outcomes. Clinical utility therefore requires a validated pathway linking the biomarker result to confirmatory evaluation, an appropriate management decision, and a clinically meaningful outcome.

**Figure 1:**
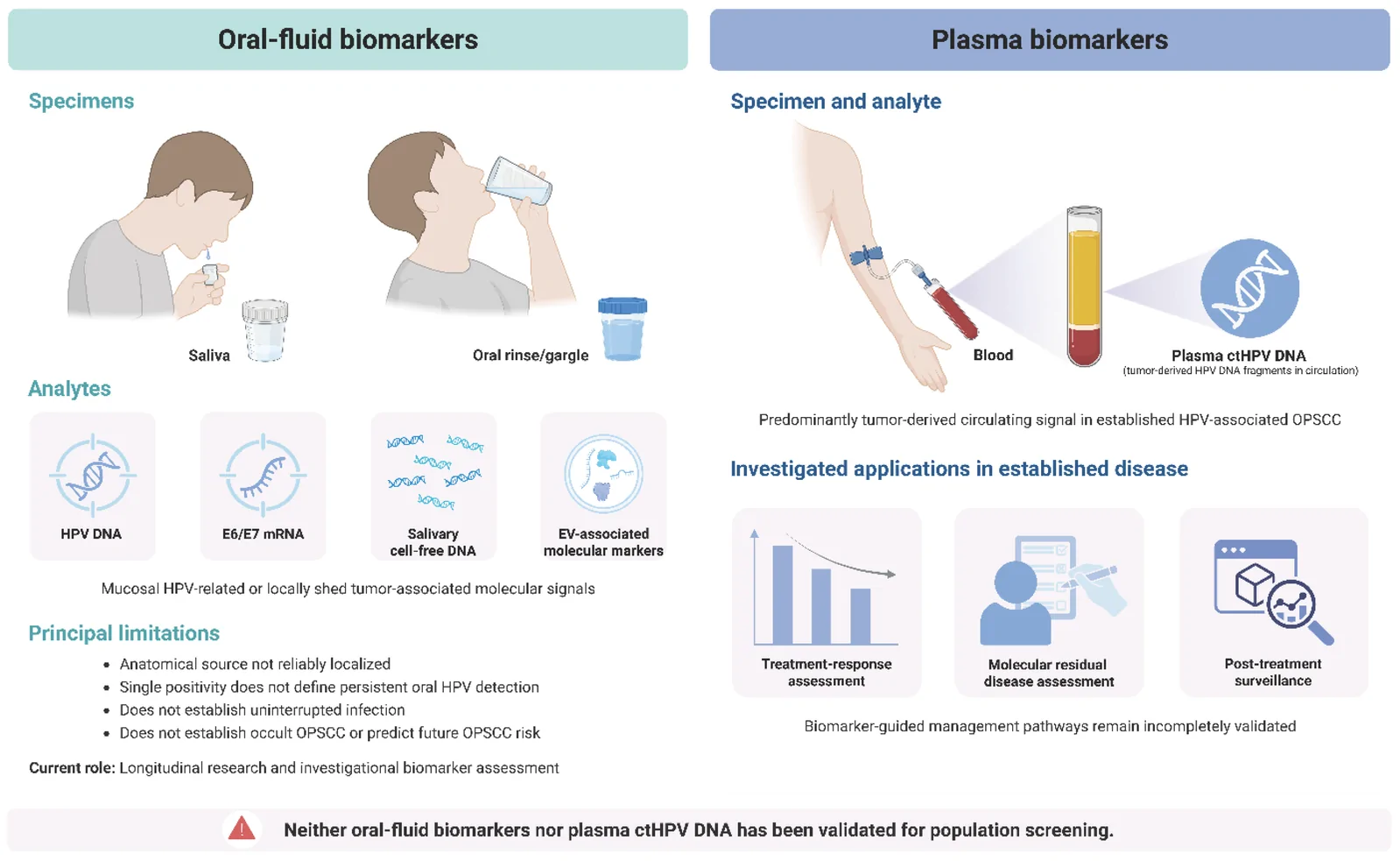
Biological and clinical interpretation of liquid-biopsy signals across clinical contexts. Oral-fluid biomarkers and plasma circulating tumor HPV DNA (ctHPV DNA) represent distinct biological signals. Serial genotype-specific oral HPV testing is an established research approach for longitudinal oral HPV assessment, whereas its clinical use for individual-level risk prediction remains unvalidated. Plasma ctHPV DNA has more advanced clinical evidence for treatment monitoring and post-treatment surveillance in patients with established HPV-associated OPSCC. Neither oral-fluid biomarker testing nor plasma ctHPV DNA has been validated for population screening. The figure was created in BioRender. Pnu, M. (2026) (https://BioRender.com/l7usebj).

Proposed multimarker risk models should consequently be evaluated for incremental predictive performance beyond serial genotype-specific oral HPV assessment and a prespecified set of relevant clinical risk factors. Evaluation should include discrimination, calibration, external validation, applicability to the intended-use population, and clinical actionability rather than analytical detectability alone [6,42]. Figure 1 summarizes the distinct biological sources, clinical applications, and interpretive limitations of oral-fluid biomarkers and plasma ctHPV DNA.

## 6. Candidate and Emerging Adjunct Biomarkers

### 6.1. Host and Viral DNA Methylation Biomarkers

DNA methylation is being investigated as an adjunct biomarker in HPV-associated OPSCC, but the evidence reviewed here derives predominantly from patients with established cancer rather than prospective longitudinal cohorts of individuals without known disease. In tissue, Birknerova et al. reported differential methylation of *WT1*, *PAX6*, *CADM1*, and *RARβ* between OPSCC and nonmalignant controls. *CADM1* and *WT1* hypermethylation was also more frequent in HPV-positive than HPV-negative tumors [43]. The transferability of methylated DNA markers from HPV-positive cervical squamous cell carcinoma to HPV-associated OPSCC appears limited. Among 21 methylated DNA markers previously validated in HPV-positive cervical squamous cell carcinoma, 18 (86%) achieved an area under the curve of at least 0.90 in cervical cancer, compared with five (24%) in HPV-associated OPSCC. Nevertheless, 19 (90%) discriminated HPV-associated OPSCC from nonmalignant tonsil controls better than chance [44]. These findings indicate that methylation marker performance varies by anatomical site and that markers validated in cervical cancer cannot be assumed to retain equivalent discriminatory performance in OPSCC.

Salivary viral DNA methylation is more directly relevant to noninvasive biomarker development, although current evidence remains preliminary. In pretreatment saliva samples from 18 patients with HPV-associated head and neck cancer and 10 HPV-positive controls, four HPV16 CpG sites in the *L1* and *L2* genes showed significant differences in methylation [45]. However, this small cross-sectional study was not restricted to OPSCC and did not evaluate serial type-specific HPV detection or subsequent cancer development. Overall, host and viral DNA methylation should currently be regarded as an exploratory approach for molecular characterization and biomarker development, with limited direct evidence and no prospective validation for longitudinal risk prediction in cancer-free populations.

### 6.2. HPV Serological and Immune Biomarkers

Among serological biomarkers, HPV16 E6 antibodies have the strongest evidence as a candidate prediagnostic marker of future oropharyngeal cancer risk, but they reflect a systemic host response rather than contemporaneous oral HPV detection. Using a risk prediction model that integrated data from nine prospective cohorts with U.S. population data on risk factors, cancer incidence, and mortality, Robbins et al. estimated 10-year oropharyngeal cancer risks of 17.4% (95% CI, 12.4–28.6) at age 50 years and 27.1% (95% CI, 19.2–45.4) at age 60 years among HPV16 E6-seropositive men. However, these were model-based estimates, and an appropriate surveillance protocol after a positive result has not been established [46]. In 3997 cancer-free men, HPV16 E6 seropositivity was rare (0.35%) but generally stable once detected, whereas oral HPV16 DNA positivity was not persistent during follow-up [47]. HPV16 E6 serology should therefore be regarded as a candidate prediagnostic risk marker rather than a measure of contemporaneous or persistent oral HPV16 detection.

Evidence from salivary antibodies and intratumoral cellular immune responses addresses distinct clinical questions. In a longitudinal study of 39 women, including 13 with study-defined persistent oral HPV16 DNA detection for at least 12 months, salivary HPV16 L1 antibody levels fluctuated over follow-up and did not differ between participants with persistent and transient detection [48]. In contrast, the combined presence of intratumoral T cells specific for HPV16 E2, E6, and E7 was associated with superior survival in patients with established HPV-associated OPSCC, supporting their relevance to tumor immunity and prognosis rather than longitudinal oral HPV assessment [49]. Overall, current evidence on salivary antibodies and intratumoral HPV-specific immune responses addresses biological and prognostic questions that are distinct from prediagnostic serological risk stratification.

### 6.3. Microbiome and Virome Profiling

The oral microbial ecosystem includes bacteria, fungi, archaea, and viruses, including bacteriophages [50,51]. Oral microbial dysbiosis and interactions between microbes and the host may influence local inflammatory and immune processes, epithelial barrier integrity, and tumor biology [50,51,52]. However, features of microbial communities and the broader oral virome have not been established as specific markers of persistent type-specific oral HPV detection. Oral microbiome profiles have been associated with contemporaneous high-risk oral HPV detection, although the observed associations have varied across study populations. In a population-based cross-sectional analysis of 5496 adults, lower bacterial richness and phylogenetic diversity were modestly associated with high-risk oral HPV detection [53]. In a separate study of 63 cancer-free men, bacterial richness was significantly higher among high-risk HPV-positive participants without HIV, whereas no significant differences in microbial diversity were observed among participants with HIV [54]. Direct comparison is limited by differences in population characteristics, sampling framework, HIV stratification, and analytical methods. Although participants in the latter study were selected from a longitudinal cohort, the microbiome analysis was cross-sectional and evaluated prevalent high-risk HPV status. It therefore could not determine whether microbial differences preceded HPV acquisition, contributed to persistent type-specific detection, or resulted from contemporaneous HPV-associated changes in the oral environment.

Prediagnostic evidence provides temporal but not HPV-specific support. In a nested case–control study using oral wash specimens collected before diagnosis, selected bacterial species and a microbial risk score comprising 22 bacteria were associated with subsequent HNSCC development [55]. However, the endpoint included cancers of multiple head and neck subsites, HPV16-negative participants predominated, and the findings did not establish a microbial signature specific to HPV-associated OPSCC or persistent oral HPV detection. Oral virome research remains comparatively underdeveloped [50], and no reproducible virome signature for persistent oral HPV detection or incident HPV-associated OPSCC was identified in the literature reviewed here. Microbiome and virome features should therefore be regarded as exploratory adjuncts whose incremental predictive value requires prospective evaluation in populations representative of the intended use.

### 6.4. Proteomic and Metabolomic Profiling

Clinical proteomic and metabolomic evidence reviewed here derives mainly from patients with established cancer and has focused on tumor biology and prognosis rather than longitudinal oral HPV assessment [56,57]. In a tissue-based analysis of 15 HPV-positive HNSCC tumors, recurrence-associated proteomic and phosphoproteomic patterns included extracellular matrix remodeling, enrichment of suppressive myeloid features, and reduced T-cell-associated signals [57]. However, the recurrence group included three primary tumors collected before subsequent recurrence and four recurrent tumors obtained during salvage surgery after relapse. The observed differences therefore cannot be interpreted as a purely pretreatment prognostic signature and primarily characterize tumor biology associated with recurrence rather than establish a noninvasive biomarker for persistent oral HPV detection.

**Figure 2:**
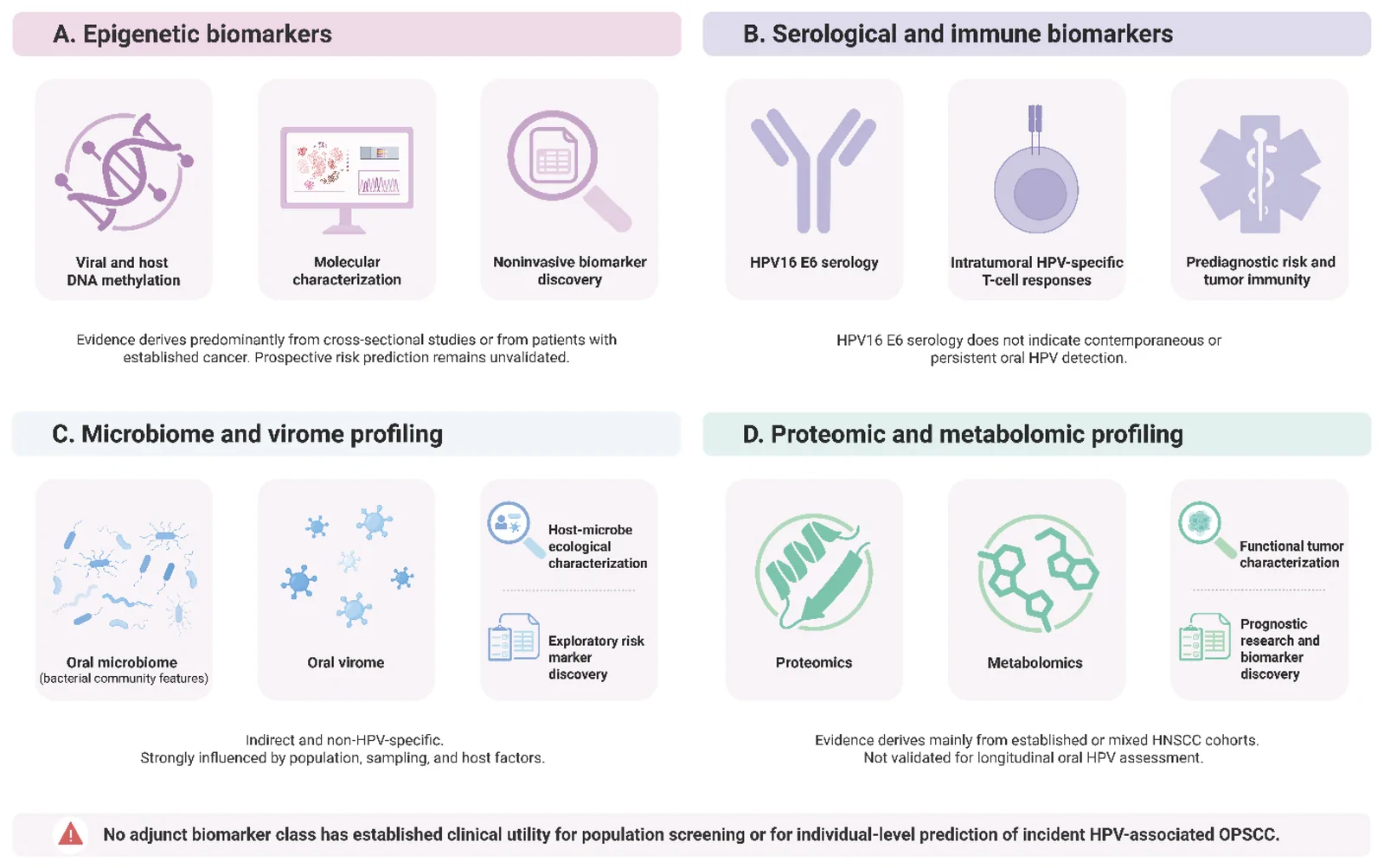
Candidate and emerging adjunct biomarkers relevant to longitudinal oral HPV assessment and HPV-associated OPSCC. Epigenetic, serological and immune, microbiome and virome, and proteomic and metabolomic approaches provide complementary biological information but differ substantially in clinical context, biological specificity, and evidence maturity. HPV16 E6 serology provides prediagnostic risk information but does not measure contemporaneous or persistent oral HPV detection. The remaining approaches shown are largely exploratory or discovery-stage, and none has established clinical utility for population screening or individual-level prediction of incident HPV-associated OPSCC. The figure was created in BioRender. Pnu, M. (2026) (https://BioRender.com/gw031nc).

Circulating metabolomic evidence is similarly exploratory. Pretreatment plasma metabolomic profiling of 209 patients with HNSCC identified two metabolic subtypes. Among ever-smokers, subtype A was associated with poorer overall and progression-free survival than subtype B after adjustment for HPV status and other clinical factors [56]. However, the cohort was not restricted to HPV-associated OPSCC, and the study did not identify an HPV-specific metabolic signature or evaluate persistent oral HPV detection or incident OPSCC. Current proteomic and metabolomic evidence should therefore be regarded as discovery-stage evidence for tumor characterization and prognostic research, with only indirect relevance to longitudinal oral HPV assessment and prediagnostic OPSCC risk prediction. Figure 2 summarizes the principal adjunct biomarker classes, their potential contributions, and their current evidentiary limitations.

## 7. Emerging Discovery Technologies and Integrated Models

### 7.1. Single-Cell and Spatial Molecular Profiling

Single-cell RNA sequencing and spatial profiling provide high-resolution characterization of malignant, immune, and stromal heterogeneity in tumor tissue. In HPV-stratified HNSCC, including HPV-associated OPSCC, these approaches have identified distinct immune-cell states and spatially organized immune and stromal niches [58,59,60]. HPV DNA in situ hybridization identified intratumoral heterogeneity of HPV DNA status in a subset of HPV-associated OPSCCs. Spatial transcriptomics was then used to characterize associated tumor-cell states and microenvironmental features. These findings characterize heterogeneity in established tumors but do not validate HPV DNA heterogeneity as a marker of persistent oral HPV detection or prediagnostic OPSCC risk [61]. These approaches should therefore be regarded as discovery-stage tools for tumor characterization and candidate biomarker generation, while evidence supporting their use in noninvasive longitudinal oral HPV assessment remains limited and indirect.

### 7.2. Multi-Omics Integration and Biological Characterization

Multi-omics approaches integrate complementary molecular and clinicopathological data to characterize tumor heterogeneity and identify candidate biomarkers. In HNSCC, integrated analyses of genomic, transcriptomic, microRNA, proteomic, and histopathological features have identified molecular correlates of tumor phenotypes, although several findings have been more extensively characterized in HPV-negative than HPV-positive disease [62]. In HPV-associated OPSCC, such approaches may improve biological characterization of tumor heterogeneity and support candidate biomarker discovery, but current evidence derives predominantly from established tumors rather than prospective longitudinal assessment before cancer diagnosis [63]. Multi-omics should therefore be regarded as a discovery-stage framework for tumor characterization and candidate biomarker generation, while current evidence provides only indirect support for its relevance to longitudinal oral HPV assessment and prediagnostic OPSCC risk prediction.

### 7.3. Computational Methods and Artificial Intelligence

Artificial intelligence (AI) is relevant to this review as an emerging computational framework for extracting and integrating complex tumor-derived data rather than as an HPV biomarker itself. In established HPV-associated OPSCC, AI-based computational pathology has been used to derive prognostic features from histopathological images, while multimodal deep learning has integrated radiological and pathological information for outcome prediction [64,65]. Current AI applications in the literature reviewed here address tumor characterization and prognosis after cancer diagnosis and have not been validated for prediagnostic OPSCC risk prediction or longitudinal oral HPV assessment. Clinical translation of prediction models will require transparent reporting of data sources, intended use, model development, and performance evaluation, including discrimination and calibration, followed by independent validation in clinically representative populations [42].

### 7.4. Toward Translational Multimodal Risk Models

Serial genotype-specific oral HPV testing provides a pragmatic longitudinal measure of repeated type-specific detection [6,9] and may serve as a reference component for evaluating the incremental value of future multimodal risk models. HPV16 E6 serology provides complementary prediagnostic information, whereas evidence for most other adjunct and emerging biomarker approaches reviewed here derives from studies of established cancer or from discovery-stage research and provides limited direct support for prediagnostic risk assessment [46]. Within the literature reviewed here, integrated models combining serial oral HPV measures with adjunct biomarkers have not undergone prospective performance evaluation for incident HPV-associated OPSCC. Translational models should therefore be developed and evaluated in clinically representative cancer-free populations, with a prespecified intended use, prespecified endpoints, and assessment of incremental predictive performance, discrimination, calibration, and clinical actionability [6,42]. The relative clinical maturity and current readiness of the sampling and biomarker approaches reviewed here are summarized in Table 3.

## 8. Discussion and Conclusions

The principal challenge in longitudinal oral HPV assessment is not analytical detection alone but determining whether a detected signal has biological and clinical significance. Repeated detection of the same high-risk HPV genotype in serial oral specimens provides a pragmatic measure of persistent oral HPV detection, but it does not establish uninterrupted infection, sustained viral oncogene transcription, malignant transformation, or individual-level risk of future HPV-associated OPSCC. Estimates of persistence and clearance are also sensitive to sampling interval, operational definition, and study population, limiting direct comparison across longitudinal studies [9]. Improvements in analytical sensitivity or detection yield should therefore not be interpreted as evidence of clinical validity or screening utility. No validated population-based screening strategy for HPV-associated OPSCC is currently available [6]. Population screening should also be distinguished from future targeted risk-stratification approaches. The absence of a validated population-screening strategy does not exclude the possibility that selected higher-risk populations could ultimately benefit from more targeted assessment. However, such approaches would require prospective validation in populations representative of their intended use, with prespecified eligibility criteria, endpoints, and management pathways. This distinction is particularly important because the evidence reviewed here derives from heterogeneous populations, including male-only cohorts, populations enriched for people living with HIV, geographically distinct cohorts, cancer-free individuals, and patients with established HNSCC or OPSCC. Findings from these populations should not be assumed to be directly transportable across sex, immune status, geographic setting, disease prevalence, or disease stage. Future validation should therefore report performance across clinically relevant subgroups rather than rely solely on aggregate estimates.

Interpretation must also remain specific to specimen source, biological target, and clinical context. Oral rinse and gargle provide practical broad-field specimens for repeated HPV assessment, whereas current evidence does not establish that additional site-directed sampling improves longitudinal oral HPV assessment or prediagnostic OPSCC risk prediction despite identifying additional HPV-positive findings in cross-sectional studies [20]. Tumor-tissue HPV testing addresses HPV attribution in established carcinoma and should not be extrapolated to oral screening. Similarly, plasma ctHPV DNA is a tumor-derived biomarker studied primarily for treatment response assessment, molecular residual disease assessment, and post-treatment surveillance in established HPV-associated OPSCC rather than for defining persistent oral HPV detection [31]. Maintaining these distinctions between mucosal HPV detection, viral oncogene transcription, and tumor-derived HPV signals is necessary to avoid attributing clinical meaning that the underlying biomarker cannot support. Plasma ctHPV DNA illustrates the distinction between biomarker performance and clinical utility. Prospective studies support its potential role in post-treatment surveillance, and in some patients a detectable biomarker signal may precede clinically confirmed recurrence [31]. However, earlier biomarker detection should not be equated with demonstrated improvement in patient outcomes. Clinical utility requires a validated pathway linking a biomarker result to confirmatory evaluation, an appropriate management decision, and a clinically meaningful outcome. This distinction also applies to biomarker-informed treatment adaptation, for which feasibility does not by itself establish comparative effectiveness or routine clinical benefit. Prolonged detectable results without documented disease and undetectable results at the time of confirmed recurrence further emphasize the need to evaluate the consequences of testing as well as its analytical or predictive performance.

Among potential adjunct biomarkers, HPV16 E6 serology provides notable prediagnostic risk information, whereas most other molecular, immune, microbiome, proteomic, metabolomic, single-cell, spatial, multi-omics, and AI-based approaches reviewed here remain supported mainly by cross-sectional studies, selected cancer cohorts, or investigations of established disease. These approaches may contribute to biological characterization and candidate biomarker discovery, but their relevance to prediagnostic risk assessment cannot be assumed. Evidence generated in clinically selected cancer populations has only indirect relevance to cancer-free populations targeted for longitudinal oral HPV assessment or prediagnostic OPSCC risk prediction, and apparent biomarker performance may not be preserved when transferred to populations with different baseline disease prevalence or clinical characteristics. A central translational question is therefore whether an adjunct biomarker or integrated model adds predictive information beyond serial genotype-specific oral HPV assessment and a prespecified set of relevant clinical risk factors. Future models should be evaluated in clinically representative cancer-free populations that reflect their intended use, with prespecified endpoints and assessment of discrimination, calibration, incremental predictive performance, external validation, and clinical actionability [6,42]. Analytical detectability alone is insufficient. Models should also be evaluated for their potential consequences in practice, including unnecessary investigations after positive results, false reassurance after negative results, repeated follow-up burden, and the feasibility of implementation across different healthcare settings.

Future research should proceed in a staged manner. First, longitudinal studies should standardize oral sampling, genotype-specific testing, sampling intervals, and operational definitions of persistence and clearance. Second, the relationship between repeated type-specific HPV detection and clinically meaningful outcomes should be established in representative cancer-free cohorts, with pathologically confirmed incident HPV-associated OPSCC as the relevant disease endpoint. Third, candidate adjunct biomarkers should be evaluated for incremental predictive value beyond prespecified baseline risk models rather than for analytical detectability alone. Fourth, externally validated risk models should be linked to predefined confirmatory and management pathways. Finally, biomarker-guided strategies should be compared with an appropriate usual-care pathway using prespecified patient-important outcomes, with net clinical benefit assessed in light of unnecessary investigations and the burden of repeated follow-up.

This review has limitations. PubMed was the sole bibliographic database searched, so relevant studies indexed exclusively elsewhere may not have been captured. The review was narrative rather than systematic, and study selection was based on relevance to the biological and clinical questions addressed rather than a formal systematic-review screening process. The evidence was also heterogeneous in study population, specimen type, sampling interval, assay platform, definitions of persistence and clearance, and clinical endpoints, limiting direct comparison across studies. In addition, much of the evidence for emerging biomarkers derives from established HNSCC, mixed disease cohorts, or tumor tissue rather than prospective cancer-free populations. These differences represent not only heterogeneity in evidence maturity but also limitations in the directness and transportability of the available evidence. Conclusions regarding their relevance to persistent oral HPV detection and prediagnostic OPSCC risk should therefore be interpreted within these evidence constraints.

In conclusion, persistent oral HPV detection should currently be regarded as a longitudinal research endpoint rather than a diagnosis or validated screening result. Serial genotype-specific oral HPV testing provides a pragmatic framework for studying repeated detection, while viral transcription, serological markers, and other adjunct biomarkers may provide complementary biological or risk information. Current evidence reviewed here has not established a biomarker or integrated model with validated clinical utility for population screening or individual-level prediction of incident HPV-associated OPSCC. Progress toward clinical translation will require standardized longitudinal assessment, validation in representative intended-use populations, evidence that additional biomarkers improve prediction beyond prespecified baseline risk factors, and clinically actionable management pathways that demonstrate an acceptable balance of benefits and harms. 

## Figures and Tables

**Table 1:** Study-specific operational definitions and analytical approaches in longitudinal oral HPV studies.

Study	Population and Oral HPV Assessment	Specimen and Follow-Up	Study-Specific Persistence/Clearance Definition or Analytical Approach	Key Methodological Implication
[8]	Adults living with HIV and at-risk HIV-uninfected adults enrolled in a longitudinal study of oncogenic oral HPV persistence and clearance	Oral rinse and gargle; every 6 months for 4 years in POPS, with one additional MOUTH study visit in a subset after a median 2.5-year gap	Type-specific analysis; the primary clearance definition was two consecutive negative tests. At the delayed MOUTH visit, a single negative result was considered evidence of clearance for that analysis.	Shows how clearance classification can depend on both the negative-test criterion and the structure of longitudinal follow-up.
[14]	Men with prevalent high-risk oral HPV at baseline in the multinational HPV Infection in Men (HIM) study	Oral gargle; visits approximately every 6 months, with persistence assessed at 6, 12, 18, and 24 months	Persistence of an individual HPV genotype was defined as detection of the same genotype at two or more consecutive visits. In rare cases, a single intervening genotype-specific negative result was retained within the persistence classification.	Shows how the handling of intermittent negative results can affect persistence classification.
[15]	College-age and older adults of both sexes from the Ann Arbor, Michigan community	Self-collected saliva samples using Scope mouthwash or an Oragene RE-100 kit; every 3–4 months for 3 years	Genotype-specific HPV-positive and HPV-negative states were analyzed using a continuous-time Markov multistate transition model to estimate incidence and clearance rates.	Frequent sampling and model-based estimation complicate direct comparison with studies using longer sampling intervals and visit-based clearance rules.
[16]	Adults living with HIV and HIV-negative healthy volunteers in a longitudinal study of oral HPV prevalence, persistence, and clearance	Saliva; every 3–8 months over 2 years	Persistence was defined as detection of the same HPV genotype at two consecutive visits, regardless of the interval between them; incidence and clearance were estimated using multistate transition models.	Highlights that persistence defined by consecutive visits does not necessarily represent a fixed duration.
[9]	Adults living with or at risk for HIV in a longitudinal study of oncogenic oral HPV clearance and persistence	Oral rinse and gargle; every 6–12 months for up to 10 years	Four clearance definitions were compared: one, two, or three consecutive negative tests, or negativity at the final two visits.	Median estimated time to clearance ranged from 0.68 to 2.43 years across the four definitions, showing that clearance estimates are sensitive to the operational definition used.
[17]	Men with incident or prevalent oncogenic oral HPV infection in the multinational HIM study	Oral gargle; collected approximately every 6 months; median follow-up 44.8 months	Incident and prevalent genotype-specific infections were analyzed separately. Clearance was defined as two consecutive negative tests for the specific HPV genotype. Persistence was defined as type-specific HPV detection at one or more sequential visits. Clearance probabilities were estimated using Kaplan–Meier analysis.	Distinguishing incident from prevalent detection materially affects interpretation of HPV natural history because the duration of prevalent infection before baseline is unknown; prevalent infections showed a lower probability of clearance than incident infections. This analysis draws from the same HIM study oral cohort as Bettampadi et al. (2021) [14] and should not be interpreted as independent cohort replication.

**Note:** Persistence and clearance criteria are study-specific operational definitions and do not necessarily reflect uninterrupted biological persistence or complete viral clearance. Cross-study estimates should therefore be interpreted in light of differences in study population, specimen type, sampling interval, genotype-specific assessment, handling of intermittent negative results, and statistical approach. Both analyses drew on oral HPV data from the same multinational HIM study cohort and should not be interpreted as independent cohort replication [14,17]. **Abbreviations:** HIM, HPV Infection in Men; HIV, human immunodeficiency virus; HPV, human papillomavirus; MOUTH, Men and Women Understanding Throat HPV; POPS, Persistent Oral Papillomavirus Study.

**Table 2:** Representative plasma ctHPV DNA assays evaluated in HPV-associated OPSCC: characteristics, reported performance, and development status.

Assay/Platform	Analytical Approach and HPV Coverage	Laboratory/Development Status Reported in the Cited Literature	Evidence Context and Selected Performance	Critical Interpretation	Primary Reference
NavDx/TTMV-HPV DNA	ddPCR-based TTMV-HPV DNA analysis; HPV16, 18, 31, 33, 35	Commercial CLIA high-complexity LDT; CAP accreditation reported	Pretreatment diagnosis: sensitivity 91.5%, specificity 100% [33] Prospective surveillance: per-patient sensitivity 73%, specificity 98%; sensitivity 91% in the HPV16 subgroup with detectable pretreatment TTMV-HPV DNA [34]	Supported by some of the most extensive clinical evidence summarized here; performance varies by population, HPV genotype, pretreatment detectability, and surveillance context	[33,34,35]
HPV-SEQ	NGS-based quantitative LDT targeting HPV16/18 L1	Evaluated under CLIA/CAP requirements	Concordance with primary-tissue HPV status 41/42 (97.6%); 20/20 healthy donor samples HPV16/18 negative	Supports analytical and clinical detection in established OPSCC; concordance should not be interpreted as validated population-screening sensitivity	[36]
MyHPVscore	Multiplex ddPCR LDT; HPV16, 18, 31, 33, 35, 39	Developed in a CLIA-certified laboratory; authors reported commercial availability under a licensing agreement	LoD 15.2 positive droplets/reaction for HPV16 and 20.8 for other targeted high-risk HPV types; linearity R^2^ > 0.99	Strong analytical validation; prospective clinical validity and clinical utility for surveillance or screening were not established by the validation study	[37]
CHAMP-16	Multiprobe ddPCR targeting nine HPV16 genomic regions	Investigational; patent development reported	LoD 4.1 copies/reaction; all 21 selected known HPV16-ctDNA-positive specimens were detected	21/21 detection cannot be interpreted as 100% clinical sensitivity because samples were preselected as ctDNA positive; clinical validation remains preliminary	[38]
Multiplex HPV ctDNA ddPCR assay	ddPCR; HPV16, 18, 31, 33, 35 in platelet-poor plasma	Investigational in-house assay; commercial availability not established in the publication	LoD 7.71–19.45 fragments/mL depending on genotype; *n* = 32 clinical samples: PPA 88.5%, NPA 100%, overall agreement 90.6% versus tumor p16-IHC/HPV-ISH	Primarily analytical/early clinical validation; limited genotype-specific clinical data and no clinical-outcome validation	[24]

**Note:** Reported performance measures are presented according to the terminology and clinical context of the cited studies and are not directly comparable across assays. Sensitivity/specificity, positive/negative percent agreement, concordance, and analytical limits of detection address different performance domains. Status refers to that reported in the cited literature within the review period and should not be interpreted as regulatory approval or guideline endorsement. None of these plasma ctHPV DNA assays has been validated for population screening or for defining persistent oral HPV detection in individuals without known OPSCC. **Abbreviations:** CAP, College of American Pathologists; CLIA, Clinical Laboratory Improvement Amendments; ctDNA, circulating tumor DNA; ctHPV DNA, circulating tumor HPV DNA; ddPCR, droplet digital polymerase chain reaction; HPV, human papillomavirus; IHC, immunohistochemistry; ISH, in situ hybridization; LDT, laboratory-developed test; LoD, limit of detection; NGS, next-generation sequencing; NPA, negative percent agreement; PPA, positive percent agreement; TTMV-HPV DNA, tumor tissue-modified viral HPV DNA.

**Table 3:** Clinical interpretation and maturity of sampling and biomarker approaches relevant to persistent oral HPV detection and HPV-associated OPSCC.

Specimen/Approach	Biological Target or Meaning	Principal Intended Use in the Reviewed Evidence	Main Advantages	Main Limitations	Clinical Evidence Maturity	Current Readiness	Representative References
Oral rinse/gargle with genotype-specific HPV DNA testing	Mucosal HPV DNA; repeated type-specific detection across serial specimens	Longitudinal oral HPV natural-history, persistence, and clearance research	Noninvasive and repeatable; enables longitudinal tracking of individual HPV genotypes	Does not localize the anatomical source or establish uninterrupted infection, viral oncogene transcription, malignant transformation, or individual-level future OPSCC risk	Candidate	Widely used in longitudinal research; not clinically validated for individual-level OPSCC risk prediction or population screening	[8,9,11]
Site-directed oral/oropharyngeal HPV sampling	HPV detected from a defined sampled site	Cross-sectional evaluation of anatomically targeted HPV detection	May sample additional oropharyngeal fields	Limited longitudinal evidence; access to relevant crypt epithelium uncertain; increased detection does not establish clinical validity	Exploratory	Investigational for longitudinal risk assessment	[18,20,21]
Oral-fluid E6/E7 mRNA	Viral oncogene transcription in sampled material	Biological characterization and adjunct detection in patients with known or suspected cancer	More directly reflects viral transcription than HPV DNA	Does not localize transcript source; limited longitudinal evidence; genotype coverage and assay design vary	Candidate	Investigational; not validated for persistent oral HPV assessment or screening	[10,23]
Tumor-tissue HPV testing, including p16-based and HPV-specific approaches	HPV attribution of an established carcinoma	Diagnostic evaluation of newly diagnosed OPSCC	Guideline-supported; clinically interpretable within defined pathological context	Requires tumor or cytological material; does not address persistent oral HPV detection or population screening	Established	Routine clinical use in defined diagnostic contexts	[2]
Plasma ctHPV DNA	Tumor-derived circulating HPV DNA	Treatment response, molecular residual disease research, and post-treatment surveillance in established HPV-associated OPSCC	Noninvasive systemic tumor signal; permits serial assessment	Evidence largely restricted to established cancer; isolated positive results require cautious interpretation; management pathways remain incompletely validated	Clinically advanced	Investigational/adjunctive clinical use in established HPV-associated OPSCC; not validated for population screening	[31,34,40]
HPV16 E6 serology	Systemic antibody response associated with HPV16-driven carcinogenesis	Prediagnostic risk stratification research	Can precede clinical diagnosis and provides information distinct from contemporaneous oral HPV DNA	Prediagnostic risk estimates derived largely from male cohorts; low seroprevalence in cancer-free populations; does not measure persistent oral HPV detection; no established management pathway after a positive result	Candidate	Not ready for population screening or routine risk stratification	[46,47]
Host or viral DNA methylation	Epigenetic alterations in host or HPV DNA	Tumor characterization and candidate noninvasive biomarker development	May capture molecular changes beyond HPV presence alone	Evidence mainly cross-sectional or established-cancer based; anatomical transferability is limited; prospective risk prediction unvalidated	Exploratory	Research only	[43,44,45]
Salivary cfDNA and extracellular-vesicle-associated molecular markers	Cell-free or vesicle-associated tumor/host molecular signals	Feasibility and biomarker discovery	Noninvasive and compatible with repeated sampling	Small or heterogeneous cancer cohorts; limited evidence for type-specific persistence or incident OPSCC prediction	Exploratory	Research only	[29,30]
Oral microbiome and virome profiling	Microbial-community and viral-community features	Association studies and candidate risk-marker discovery	Captures host-microbial ecological features	Indirect and non-HPV-specific; strongly influenced by population, sampling, and host factors; prospective HPV-specific validity unestablished	Exploratory	Research only	[53,54,55]
Proteomic and metabolomic profiling	Protein, signaling, and metabolic phenotypes	Tumor characterization and prognostic research	Provides functional molecular information	Evidence mainly from established or mixed HNSCC cohorts; limited HPV-associated OPSCC specificity; no longitudinal oral HPV validation	Discovery-stage	Research only	[56,57]
Single-cell, spatial, multi-omics, and AI-based approaches	High-dimensional cellular, spatial, molecular, imaging, or integrated data	Tumor characterization, biomarker discovery, and prognostic modeling	Resolves biological heterogeneity and supports multimodal integration	Evidence derives primarily from tissue- and imaging-based studies in patients with established cancer; independent external validation for prognostic applications and model development remains limited; applicability to prediagnostic risk assessment in cancer-free populations remains indirect and unvalidated	Discovery-stage	Research and model-development use; not validated for oral HPV screening or longitudinal risk prediction	[58,61,62,65]

**Note:** Clinical evidence maturity is a qualitative classification used in this narrative review and does not represent a formal GRADE assessment. The classification reflects the maturity of evidence for clinical interpretation or application rather than how commonly an approach is used in research. “Established” denotes guideline-supported clinical use within a defined indication; “clinically advanced” denotes substantial human evidence with remaining limitations in routine implementation or management pathways; “candidate” denotes human evidence supporting a biologically or clinically relevant interpretation for a specified intended use but without sufficient validation for routine clinical use; “exploratory” denotes early human association or feasibility evidence without sufficient clinical validation for the intended use; and “discovery-stage” denotes evidence directed primarily toward mechanistic characterization, biomarker discovery, or model development without sufficient clinical validation for the intended use. Analytical platforms such as qPCR, ddPCR, and NGS are not assigned independent clinical maturity because their interpretation depends on specimen type, biological target, assay design, and intended clinical use. In Table 3, evidence maturity is interpreted for the stated specimen, study population, and intended use. Applicability to other specimens, populations, or clinical purposes requires separate assessment. The use of high-dimensional data alone does not determine evidence maturity. These categories describe the maturity of evidence for the specified context and should not be interpreted as clinical practice recommendations. **Abbreviations:** AI, artificial intelligence; cfDNA, cell-free DNA; ctHPV DNA, circulating tumor HPV DNA; ddPCR, droplet digital polymerase chain reaction; GRADE, Grading of Recommendations Assessment, Development and Evaluation; HNSCC, head and neck squamous cell carcinoma; HPV, human papillomavirus; mRNA, messenger RNA; NGS, next-generation sequencing; OPSCC, oropharyngeal squamous cell carcinoma; qPCR, quantitative real-time polymerase chain reaction.

## Data Availability

No new data were created or analyzed in this study. Data sharing is not applicable to this article.
